# Supplementary material for: A blended modeling framework for real-time design and verification of safety-critical embedded systems
Source: PLoS One. 2025 Dec 4;20(12):e0337604. doi: 10.1371/journal.pone.0337604 (PMC12677560; doi:10.1371/journal.pone.0337604)
Supplement: Attachment — Submitted filename: Response to Reviewers.pdf [file pone.0337604.s002.pdf]

Rebuttal letter for the submission of Manuscript in PLOS One Journal
Manuscript Number: PONE-D-25-39470

"A blended modeling framework for real-time design and verification of safety-critical embedded systems "

Dear Editor-In-Chief,

We thank you and all reviewers for the valuable feedback. We have tried to revise the article based on the reviewer's comments and suggestions. Please find the following files:
1) Rebuttal file
2) Revised article with highlighted new changes in yellow color and
3) Revised article with new changes (no highlight).

We also submitted the latex source files of the revised article.
Sincerely,
Misbah Mehboob Awan, on behalf of all the authors.

Reviewer 1

R1.0 The manuscript presents an ambitious and relevant contribution by proposing a blended modelling framework for real-time design and verification of safety-critical embedded systems. The integration of C, System Verilog, Timed Automata, and DSML through bidirectional transformations is timely and impactful, particularly for industrial contexts such as ventilator and cruise control systems. However, while the framework shows promise, several aspects of the manuscript would benefit from deeper clarification, stronger validation, and tighter presentation. The following comments are intended to help the authors strengthen the technical rigor and readability of the paper before resubmission.

Author's response: We sincerely thank the reviewer for their thoughtful and encouraging assessment of our work. We are grateful that the reviewer recognizes the ambition, relevance, and industrial applicability of our proposed blended modeling framework, particularly its ability to integrate C, SystemVerilog, Timed Automata, and DSML through bidirectional transformations for safety-critical contexts such as ventilator and cruise control systems. We also deeply appreciate

the constructive feedback regarding the need for deeper clarification, stronger validation, and tighter presentation. These observations are invaluable in guiding us to improve both the technical rigor and the readability of the manuscript. In the revised version, we have carefully addressed each of the reviewer's detailed comments point by point, strengthening the novelty claims, enhancing the evaluation and validation components, and improving the clarity of figures, tables, and narrative flow. We believe these revisions substantially improve the overall quality and accessibility of the paper.

R1.1 The novelty of the work is promising, but the distinction from existing blended modeling frameworks such as HOT-based synchronization, EMF-based solutions, and AUTOSAR workflows is not emphasized strongly enough. A comparative table with technical differentiators would clarify the contribution.

Author's response: We sincerely thank the reviewer for this valuable observation. We fully agree that the distinction between our proposed framework and existing blended modeling approaches requires clearer articulation. Accordingly, we have substantially revised the Discussion section to include a **c**omparative analysis table (Table 13) that systematically contrasts our framework with existing blended modeling approaches.

The updated table and its accompanying narrative highlight the key differentiators of our research, including the use of a parser-based transformation engine, runtime bi-directional synchronization, and multi-notation coverage across C, SystemVerilog, UPPAAL, and DSML, capabilities not concurrently supported in existing frameworks. This enhancement strengthens the novelty, clarity, and scientific positioning of the proposed work within the broader context of blended modeling research.

R1.2 The architecture description is detailed but too descriptive; a layered workflow diagram showing data exchange across C, SystemVerilog, Timed Automata, and DSML would make the framework clearer. Complexity analysis of the transformation engine (time and space cost) should also be added.

Author's response: We sincerely thank the reviewer for this insightful suggestion. As correctly noted, the architecture description in its current form is already detailed, and introducing additional layered diagrams would risk overcrowding the figure. Importantly,

the bi-directional exchange of information across notations (C, SystemVerilog, Timed Automata, and DSML) is already visually represented in our framework diagram within the Transformation Engine and Multi-Representation Editor components, where synchronization takes place in real-time. The first stage (Language Subset Evaluation and Selection) does not involve data exchange but serves to scope the subset of constructs for transformation, and this has now been explicitly clarified in the text to avoid ambiguity. To further strengthen the manuscript, we have added a more explicit textual explanation of data exchange across notations in Section 3.1 (highlighted paragraph), ensuring clarity without compromising readability. Regarding the reviewer's suggestion for complexity analysis, we agree that this is an essential addition. Accordingly, Section 6 (Performance Evaluation) has been revised to include a complexity analysis of the Transformation Engine in terms of both time and space cost. In particular, parsing and transformation are shown to operate in linear time O(n) with respect to the size of the input program, while memory requirements for intermediate structures (AST and DSML meta-model) also scale linearly. Empirical results in Section 6 confirm this theoretical analysis, demonstrating low memory usage and transformation latency, thereby validating the scalability and efficiency of the approach. We believe these clarifications and additions directly address the reviewer's concern and significantly enhance the technical rigor of the manuscript.

R1.3 The rationale for language subset selection is qualitative. Empirical justification, such as coverage statistics of industrial code or representative benchmarks, is required. Excluding concurrency constructs like threads, tasks, and interrupts limits applicability for safety-critical systems; this requires justification and discussion of future extension.

Author's response: We sincerely thank the reviewer for this valuable comment. In response, we have extended the manuscript to strengthen the empirical justification of the selected language subsets. Specifically, we have added Section 3.3.2 (Empirical Coverage of Selected Subsets), which provides quantitative analysis of the representativeness of the chosen constructs based on industrial code benchmarks. To further support this, we have introduced Table 4 (Coverage of Selected Language Subsets in Industrial Benchmarks), which clearly demonstrates that the chosen subset provides broad coverage for typical embedded system designs. Relevant studies have been cited to substantiate this analysis (Refs. 35 and 36). Regarding the exclusion

of concurrency constructs such as threads, tasks, and interrupts, we acknowledge their importance in safety-critical contexts. To address this, we have explicitly discussed the rationale for their omission in the current phase, while outlining concrete strategies for their incorporation in subsequent work. A dedicated paragraph has been added in the Future Directions section, highlighting that extending our framework to handle concurrency constructs remains an essential next step toward broader applicability. All of these additions have been highlighted in the revised manuscript for the reviewer's convenience.

R1.4 Transformation rules need deeper discussion of semantic equivalence issues, e.g., mapping System Verilog timing constructs to Timed Automata clocks. Examples of constructs where exact mapping was not possible, and the strategies used to approximate them, should be presented. Quantitative data on information loss during reverse transformations would strengthen the claims.

Author's response: We sincerely thank the reviewer for this insightful observation. We would like to clarify that the discussion on semantic equivalence and information loss in transformation rules has already been incorporated in the manuscript under Section 3.5: Critical Analysis of Round-Trip Transformations in Blended Modeling. However, this section has now been further refined and expanded to explicitly address the reviewer's concern and enhance clarity. This section explicitly analyzes the inherent semantic mismatches that arise when mapping constructs across C, SystemVerilog, Timed Automata (UPPAAL), and DSML, and highlights the challenges of achieving exact round-trip consistency. To ensure clarity, we presented concrete examples where one-to-one mappings were not feasible, such as the handling of extern variables, type precision mismatches (uint8_t and uint32_t), define directives, and enumerations, along with the strategies adopted to approximate or preserve these constructs. Furthermore, Table 8 (Round-Trip Transformation Analysis) systematically documents these challenges, the mitigation approaches, and whether information loss occurred, thereby providing quantitative evidence of fidelity across transformations. Complementing this, Figure 5 (Round Trip Transformations) illustrates the notion of delta loss and demonstrates how information discrepancies may accumulate during round-trip conversions. To strengthen the manuscript, we have now highlighted these discussions more prominently in the revised version so that the treatment of semantic equivalence, approximation strategies,

and information loss is immediately evident to the reader. Additionally, a deeper discussion of semantic equivalence issues and approximation strategies is essential to strengthen the framework. To address this, we have expanded the manuscript by adding a comprehensive discussion of semantic evaluation issues in the Discussion section. This new paragraph explicitly details how timing constructs in SystemVerilog are mapped to UPPAAL clocks, how preprocessor directives and data types are approximated across notations, and where information loss may occur in reverse transformations. We also explain the strategies adopted, such as preserving constructs as comments, type generalization, and programmatic recovery, to minimize delta loss and maintain semantic fidelity. This addition directly addresses the reviewer's concern by clarifying cases where exact semantic equivalence cannot be achieved, while outlining how our framework mitigates such discrepancies to ensure practical round-trip transformations.

R1.5 Case studies are appropriate, but evaluation metrics are too narrow. In addition to latency, memory, and transformation accuracy, please include productivity gains (developer time saved), defects detected or prevented due to synchronization, and scalability results (lines of code or model size handled). Comparisons with baseline workflows or existing frameworks would highlight practical improvements.

Author's response: We sincerely thank the reviewer for this constructive suggestion. We fully agree that, beyond latency, memory, and transformation accuracy, broader evaluation metrics such as productivity gains, defect detection, and scalability are essential for demonstrating the practical impact of our framework. At this stage, the framework is still in its growing phase, we have now explicitly addressed this point in the revised manuscript. A new paragraph has been added to the Future Work section (Section 7.2), where we outline our plan to conduct industrial-scale validation with four independent development teams. These studies will assess productivity improvements, synchronization-driven defect prevention, and scalability across large codebases. We believe this addition acknowledges the reviewer's concern and sets a clear trajectory for the next phase of our research.

R1.6 Tool support and reproducibility need more clarity. Specify whether the tool supports round-trip editing in all notations, what dependencies (ANTLR version, UPPAAL integration) are required, and provide screenshots or GUI demonstration figures.

Author's response: Your point is right. We have included this aspect in the Limitations section. At the current stage, our framework cannot perform direct external tool collaboration with UPPAAL, C compilers, or Simulink. For this purpose, users need to manually import the generated code into the respective working environments/tools. To clarify compatibility, we have now explicitly mentioned the supported versions: UPPAAL 4.1.26, GCC 11.2 (C compiler), SystemVerilog IEEE 1800-2017, and ANTLR 4.13.0. (Section 7.3). Additionally, GUI demonstration figures have already been provided in Section 4 (Implementation Architecture). We cannot add more figures, since doing so would make the manuscript unnecessarily long and more like a user manual, a concern also raised in your subsequent comment regarding paper length. Instead, for reproducibility and further investigation, we have made all resources available in our GitHub repository (Ref 33), including the Eclipse RCP tool for direct usage and exploration.

R1.7 The manuscript is overly long. Detailed grammar rules and AST breakdowns could be moved to supplementary material, while keeping only key illustrative examples in the main text. Figures should have clearer captions explaining their relevance to the framework.

Author's response: We sincerely thank the reviewer for this valuable observation. We acknowledge that the manuscript was lengthy in its earlier version, with detailed grammar rules and complete AST breakdowns contributing to excessive technical depth in the main text. In response, we have retained only key illustrative examples while relocating full grammar specifications and AST expansions to Appendix A to enhance focus and clarity. Furthermore, we have excluded non-essential transformation rules from Section (Proof of Concept – Validation and Evaluation). Only the most representative and impactful rules are retained to emphasize conceptual clarity, maintain scientific precision, and enhance the overall coherence and conciseness of the paper. In addition to these structural refinements, the entire manuscript has been systematically revised to reduce content. Several related paragraphs have been merged into concise, cohesive discussions to improve narrative flow and scientific readability. The reduced version, with these refinements highlighted in the manuscript, now offers a more streamlined presentation without compromising technical completeness or scholarly rigor.

Furthermore, figure captions have been thoroughly revised and expanded to clearly convey their significance and linkage to the proposed framework.

R1.8 Limitations are understated. Explicitly discuss current lack of concurrency modeling, scalability to larger industrial systems, and potential challenges in heterogeneous hardware–software co-design. A short future work section would better position the research.

Author's response: We thank the reviewer for this valuable observation. In the revised manuscript, we have expanded the Limitations section 7.1 to explicitly acknowledge the current lack of concurrency modeling (threads, tasks, interrupts), the challenges of scaling the framework to very large industrial systems, and the complexities of heterogeneous hardware–software co-design. These aspects are identified as critical but currently unsupported features of our framework. Furthermore, to better position this research, we have introduced a Future Work subsection 7.2, where we outline planned extensions including (i) incorporating concurrency constructs to enhance applicability for safety-critical domains, (ii) industrial-scale validation on larger case studies to systematically assess scalability, and (iii) exploring hybrid strategies to address cross-domain hardware–software co-design challenges. These additions provide a clearer picture of the framework's present boundaries and its evolution roadmap.

R1.9 Overall, the paper has strong potential, but revisions are needed to clarify novelty, strengthen validation, and sharpen the technical depth of transformation and semantic fidelity claims.

Author's response: Overall, we sincerely appreciate the reviewer's detailed and constructive feedback. We acknowledge that while the paper has strong potential, revisions were indeed necessary to more clearly highlight the novelty of our framework, strengthen the validation with empirical evidence and case studies, and sharpen the discussion on transformation rules and semantic fidelity. We have accordingly revised the manuscript to address these points by (i) clarifying the uniqueness of our blended modeling approach, (ii) expanding validation with empirical coverage, scalability considerations, and round-trip analysis, and (iii) deepening the discussion of semantic equivalence issues and limitations. These revisions significantly enhance the technical depth, reproducibility, and positioning of our work within the broader model-driven engineering and embedded systems research

landscape.

Reviewer 2

R2.0 An in-depth analysis of the manuscript reveals a high-quality research work, characterized by clarity of presentation, methodological rigor, technical soundness, and a strong alignment between the data presented and the conclusions reached. The manuscript is presented clearly and intelligibly, written in standard technical English, consistent with scientific publications in the field of software engineering and embedded systems. The structure follows the conventional academic format, with logical sections that guide the reader from the contextualization of the problem to the presentation of the results and conclusions, facilitating understanding of the work. One of the strongest points of the work is the authors' commitment to transparency and reproducibility. They have made the complete implementation of the proposed framework fully available to promote future research. The source code, including grammars, transformation rules, "visitor" classes, and the graphical interface, is publicly accessible in a GitHub repository. Additionally, the authors have provided a precompiled executable for easy use and verification of the results without the need for compilation. The article does not perform a formal statistical analysis (with hypothesis testing, for example), which is common in other scientific fields. Instead, the authors conduct a rigorous empirical performance evaluation, which is the appropriate methodology for validating the effectiveness of a software framework like this. The evaluation was based on multiple well-defined parameters, such as transformation latency, round-trip transformation accuracy, edge case handling, memory usage, and scalability. The analysis presented is systematic and suitable for measuring the feasibility and efficiency of the proposed solution in the context of embedded systems. The manuscript is technically sound, based on well-established principles. The solution architecture employs robust, industry-standard tools and techniques, such as:

- The use of a central abstract syntax (DSML meta-model) to ensure semantic consistency across different languages.
- The use of the ANTLR tool for language parsing ensures a robust and reliable code interpretation process.

– The use of appropriate design patterns, such as the Visitor Pattern, to apply transformation rules in a modular and extensible manner.

The robustness of the work is reinforced by the transparent discussion of the technical challenges inherent to the approach, such as the inevitable loss of information in bidirectional transformations, which is analyzed in detail. More importantly, the data presented convincingly corroborate the paper's conclusions. The claims of efficiency and practicality are supported by low latency times (mostly below 130 ms for 100 lines of code) and modest memory usage. The robustness and adaptability of the framework are demonstrated by successful validation on two industrial case studies from distinct domains—a medical ventilator system and an automotive cruise control system. The main conclusion that the framework maintains semantic fidelity with minor losses is directly supported by the accuracy data, which shows hit rates above 90% for most transformation paths, while the analysis identifies and explains the exact sources of these minor losses. In conclusion, the combined analysis indicates that the manuscript is an exemplary piece of research. It is well-written, technically sound, transparent about its limitations, and, crucially, its conclusions are strongly supported by the empirical data presented.

Author's response: We sincerely thank you for the highly encouraging and comprehensive assessment of our work. We deeply appreciate the recognition of our efforts toward methodological rigor, clarity of presentation, transparency, and reproducibility. We are also grateful that the reviewer acknowledged the robustness of our architectural design, the appropriateness of our evaluation methodology, and the practicality of our framework as demonstrated through industrial case studies. The positive feedback on our empirical evaluation, semantic fidelity analysis, and open availability of all implementation artifacts motivates us to further strengthen the framework in future work. We are encouraged that the reviewer finds our manuscript to be well-written, technically sound, and exemplary in balancing transparency with rigor.

R2.1 Despite the framework's robustness, future improvements could focus on mitigating the small information losses that occur during round-trip transformations and expanding the currently supported language subsets to encompass more complex and rare code constructs. Validat-

ing the approach in larger-scale and more complex industrial projects would serve to more rigorously test its scalability and robustness in real-world scenarios. Additionally, improvements in the translation of rich temporal semantics (such as those of Timed Automata) would significantly increase its applicability and impact in industry.

Author's response: We sincerely thank the reviewer for this constructive feedback and valuable suggestions. As correctly noted, while our framework currently achieves high round-trip accuracy, certain information losses are unavoidable due to inherent semantic mismatches between languages. We agree that mitigating these losses remains an important direction for advancing the robustness of blended transformations. Accordingly, we have explicitly acknowledged this in the Limitations section and outlined future strategies for reducing such discrepancies, including hybrid semantic preservation methods, traceability annotations, and enhanced transformation heuristics. Regarding the expansion of supported language subsets, we have now clarified in Section 3.3.2 (Empirical Coverage of Selected Subsets) that our current focus is on widely used industrial constructs, while more advanced concurrency and temporal features (e.g., threads, interrupts, rich timing semantics) will be integrated in subsequent phases of the framework. To strengthen this point, we have highlighted these directions in the Future Work section 7.2, where we outline our plan to extend subset coverage and enhance support for expressive temporal constructs, particularly within Timed Automata. Finally, we concur with the reviewer's recommendation on validating scalability in larger and more complex industrial projects. While our present evaluation has focused on two representative case studies, we have included in the Future Work section 7.2 our plan to engage in broader industrial validation with multiple partners, focusing on scalability (lines of code, model size) and practical integration. We believe these revisions clearly acknowledge the reviewer's insightful observations and demonstrate how they are shaping our future research trajectory.

Reviewer 3

R3.0 This research work theme translates between multiple languages useful in embedded design. The paper is well-written and contributions are explained in a logical manner. The following comments may improve the quality of work.

Author's response: We sincerely thank the reviewer for recognizing the novelty and clarity of our work, particularly in the area of supporting translations between multiple languages for embedded system design. We appreciate the constructive feedback and have carefully addressed each of the specific comments provided below to further improve the quality, scientific depth, and overall readability of the manuscript.

R3.1 The artwork needs a revision for visibility. A few of the included pictures are barely readable.

Author's response: We thank the reviewer for this observation. We would like to clarify that all figures and application screenshots were prepared according to the official PLOS ONE journal guidelines with respect to dimensions, resolution, and quality. Nevertheless, to further enhance readability, we will carefully review the figures, adjust contrast and labeling where necessary, and ensure that the final submission provides maximum clarity for readers.

R3.2 Include a table summarizing related research outlined in section 2.

Author's response: We thank the reviewer for the observation. We have revised the Discussion section to include a structured comparative table (Table 13) contrasting our framework with existing blended modeling approaches. This table 13, along with the accompanying narrative, emphasizes the unique differentiators of our approach, namely parser-based bi-directional transformations, multi-notation synchronization (C, SystemVerilog, UPPAAL, and DSML), and so on. We believe this addition clarifies our novelty and strengthens the overall contribution of the work.

R3.3 Consider defining: Design, verification, validation, modeling, etc. Abbreviations first appeared in text should be in full term.

Author's response: We thank the reviewer for the observation. While fundamental terms such as design, validation, verification, and modeling are well-established in the embedded systems and model-driven engineering literature and therefore not defined in detail to avoid redundancy, we have ensured that all domain-specific abbreviations and technical acronyms are expanded upon first use in the revised manuscript to maintain clarity for readers from diverse backgrounds.

R3.4 It is not clear from the text whether the author implemented the code translation for the full application code or just a part of it.

Author's response: Thank you for pointing this out. To clarify, our implementation covered the full application logic of both case studies (medical ventilator and automotive cruise control), rather than isolated code fragments. However, since industrial systems are very large, we focused on complete representative applications that capture all relevant control logic and constructs necessary for verification. This ensures that the evaluation is realistic while remaining manageable. We have clarified this in the revised manuscript.

R3.5 Discussion section is very brief.

Author's response: We appreciate this observation. In the revised manuscript, we have expanded the Discussion section 7 to provide a deeper analysis of the results. Specifically, we now:

- Highlight the implications of round-trip transformation accuracy and minor semantic losses.
- Compare our approach with related transformation frameworks to emphasize novelty.
- Discuss practical limitations, such as scalability, concurrency modeling, and heterogeneous hardware–software co-design challenges.
- Provide future research directions, including support for richer temporal semantics and validation on larger industrial systems.

This expanded discussion now provides a more comprehensive reflection on the significance, limitations, and future applicability of the proposed framework.

R3.6 Traces of using GenAI needs to be removed from the paper, "This rule-based format allows clear mapping between grammar components and transformation logic in C-to-other notation conversions. Let me know if you need a matching SystemVerilog target grammar or transformation rules next."

Author's response: We thank the reviewer for pointing this out. The mentioned phrase was inadvertently left from our internal drafting notes and does not belong to the scientific content of the paper. In the revised version, we have removed all such traces and ensured that the text adheres to the standards of formal academic writing. The affected sentence has been rewritten to maintain technical clarity while avoiding any conversational or non-scholarly expressions.

R3.7 Another major change which needs to be addressed is to revise the design. It mostly consists of documents and the code is not a primary part of it. Once, the design is finalized; appropriate language (or combination) may be used for application coding. The coding is subjected to verification (testing).

Author's response: We thank the reviewer for the observation. The framework indeed emphasizes design-level models (abstract syntax trees, grammars, and meta-models) as the primary artifacts, consistent with the principles of model-driven engineering (MDE). In this paradigm, models capture both structure and semantics, while code is systematically generated and transformed from these models. Thus, the design is not limited to documents but is operationalized through transformations that produce executable C, SystemVerilog, and Timed Automata representations. To avoid ambiguity, we have revised the manuscript to explicitly state that the framework treats models as the central design element and code as derived outputs subjected to verification and testing, ensuring correctness and consistency across heterogeneous notations.

R3.8 The above comments may improve the quality of research work.

Author's response: We sincerely thank the reviewer for their constructive comments and suggestions. We have carefully addressed each point, and we believe the revisions have significantly improved the clarity, quality, and rigor of our research work. We greatly appreciate the reviewer's time and guidance in enhancing the manuscript.

Reviewer 4

R4.0 This work proposes a blended modeling framework that provides bidirectional automatic conversion between different representations (C, SystemVerilog, Timed Automata, DSML) for the design and verification processes of embedded systems. Its strength lies in its real-time synchronization between the different representations and its demonstration on two industrial examples: a ventilator and cruise control. Furthermore, performance analyses demonstrate that the conversions operate with low latency and reasonable memory consumption.

Author's response: We sincerely thank the reviewer for the positive evaluation of our work. We appreciate the recognition of the

strengths of our blended modeling framework, including its bidirectional automatic conversions, real-time synchronization, and efficient performance demonstrated on industrial case studies. The encouraging feedback motivates us to further refine and extend the framework in future work.

R4.1 First of all, the study appears to be quite long, which makes it difficult to read. It also gives the impression of being a book chapter rather than an article. Therefore, the authors should address this issue. Perhaps some sections could be included in the Appendix section.

Author's response: We sincerely thank the reviewer for this valuable observation. We acknowledge that the manuscript was lengthy in its earlier version, with detailed grammar rules and complete AST breakdowns contributing to excessive technical depth in the main text. In response, we have retained only key illustrative examples while relocating full grammar specifications and AST expansions to Appendix A to enhance focus and clarity. Furthermore, we have excluded non-essential transformation rules from Section 5 (Proof of Concept – Validation and Evaluation). Only the most representative and impactful rules are retained to emphasize conceptual clarity, maintain scientific precision, and enhance the overall coherence and conciseness of the paper. In addition to these structural refinements, the entire manuscript has been systematically revised for content reduction. Several related paragraphs have been merged into concise, cohesive discussions to improve narrative flow and scientific readability. The reduced version, with these refinements highlighted in the manuscript, now offers a more streamlined presentation without compromising technical completeness or scholarly rigor.

R4.2 The scope of the case studies appears to be limited and supplementation with larger/complex industrial systems could have strengthened the generalizability of the method.

Author's response: We sincerely thank the reviewer for this constructive feedback. Regarding the scope of case studies, we have clarified in Section 3.3.2 (Empirical Coverage of Selected Subsets) that our current focus is on widely used industrial constructs. More advanced concurrency and temporal features (e.g., threads, interrupts, rich timing semantics) will be integrated in future phases of the framework. We have highlighted these directions in the Future Work 7.2 section, including plans to extend subset coverage and enhance support for

expressive temporal constructs, particularly within Timed Automata.

We concur with the reviewer's recommendation on validating scalability in larger and more complex industrial projects. While our present evaluation focuses on two representative case studies, we have included plans in the Future Work section 7.2 to engage in broader industrial validation with multiple partners, assessing scalability (lines of code, model size) and practical integration.

R4.3 Furthermore, no comparative evaluation of the framework with existing industry standards (e.g., AUTOSAR, Simulink) has been conducted, making it difficult to clarify the practical advantages of the proposed approach.

Author's response: We thank the reviewer for the observation. We have revised the Discussion section to include a structured comparative table (Table 13) contrasting our framework with existing blended modeling literature. This table 13, along with the accompanying narrative, emphasizes the unique differentiators of our approach, namely parser-based bi-directional transformations, multi-notation synchronization (C, SystemVerilog, UPPAAL, and DSML), and so on. We believe this addition clarifies our novelty and strengthens the overall contribution of the work.

R4.4 Reference 33 cannot be used in an academic study.

Author's response: We thank the reviewer for the comment. Reference 33 points to our GitHub repository, which provides direct access to the source code, case studies, and the RCP of the project. We include this reference to ensure transparency and reproducibility of our results, allowing other researchers to examine and replicate the implementations presented in the manuscript.

R4.5 The discussion section should also compare the study with the literature and highlight its strengths and weaknesses. This will help transition from this point to the future vision more accurately. It would be even better if a summary table and commentary could be provided here.

Author's response: We thank the reviewer for the suggestion. As addressed in response to comment R4.3, we have incorporated a detailed comparison of our study with relevant literature, highlighting the framework's strengths and limitations. Additionally, we have included a summary table and accompanying commentary in the Dis-

cussion section to provide a clear transition toward the future vision and planned enhancements of the framework.

R4.6 The values given in tables 9, 10 and 11 in Section 6 need to be explained along with their reasons.

Author's response: We thank the reviewer for this valuable comment. We have revised Section 6 to provide detailed explanations of the values presented in Tables 9, 10, and 11, along with the underlying reasons and contributing factors. These clarifications help readers understand the results more thoroughly and contextualize the performance metrics within the framework's design and implementation choices.
