# Supplementary material for: A blended modeling framework for real-time design and verification of safety-critical embedded systems
Source: PLoS One. 2025 Dec 4;20(12):e0337604. doi: 10.1371/journal.pone.0337604 (PMC12677560; doi:10.1371/journal.pone.0337604)
Supplement: Attachment — Submitted filename: Review Comments .doc [file pone.0337604.s001.doc]

Reviewer Comments

The manuscript presents an ambitious and relevant contribution by proposing a blended modelling framework for real-time design and verification of safety-critical embedded systems. The integration of C, System Verilog, Timed Automata, and DSML through bidirectional transformations is timely and impactful, particularly for industrial contexts such as ventilator and cruise control systems. However, while the framework shows promise, several aspects of the manuscript would benefit from deeper clarification, stronger validation, and tighter presentation. The following comments are intended to help the authors strengthen the technical rigor and readability of the paper before resubmission.

1. The novelty of the work is promising, but the distinction from existing blended modeling frameworks such as HOT-based synchronization, EMF-based solutions, and AUTOSAR workflows is not emphasized strongly enough. A comparative table with technical differentiators would clarify the contribution.
2. The architecture description is detailed but too descriptive; a layered workflow diagram showing data exchange across C, SystemVerilog, Timed Automata, and DSML would make the framework clearer. Complexity analysis of the transformation engine (time and space cost) should also be added.
3. The rationale for language subset selection is qualitative. Empirical justification such as coverage statistics of industrial code or representative benchmarks is required. Excluding concurrency constructs like threads, tasks, and interrupts limits applicability for safety-critical systems; this requires justification and discussion of future extension.
4. Transformation rules need deeper discussion of semantic equivalence issues, e.g., mapping System Verilog timing constructs to Timed Automata clocks. Examples of constructs where exact mapping was not possible, and the strategies used to approximate them, should be presented. Quantitative data on information loss during reverse transformations would strengthen the claims.
5. Case studies are appropriate, but evaluation metrics are too narrow. In addition to latency, memory, and transformation accuracy, please include productivity gains (developer time saved), defects detected or prevented due to synchronization, and scalability results (lines of code or model size handled). Comparisons with baseline workflows or existing frameworks would highlight practical improvements.
6. Tool support and reproducibility need more clarity. Specify whether the tool supports round-trip editing in all notations, what dependencies (ANTLR version, UPPAAL integration) are required, and provide screenshots or GUI demonstration figures.
7. The manuscript is overly long. Detailed grammar rules and AST breakdowns could be moved to supplementary material, while keeping only key illustrative examples in the main text. Figures should have clearer captions explaining their relevance to the framework.
8. Limitations are under-stated. Explicitly discuss current lack of concurrency modeling, scalability to larger industrial systems, and potential challenges in heterogeneous hardware–software co-design. A short future work section would better position the research.

Overall, the paper has strong potential, but revisions are needed to clarify novelty, strengthen validation, and sharpen the technical depth of transformation and semantic fidelity claims.
